# Does Therapy with Glucagon-like Peptide 1 Receptor Agonists Have an Effect on Biochemical Markers of Metabolic-Dysfunction-Associated Steatotic Liver Disease (MASLD)? Pleiotropic Metabolic Effect of Novel Antidiabetic Drugs in Patients with Diabetes—Interventional Study

**DOI:** 10.3390/ph16091190

**Published:** 2023-08-22

**Authors:** Marcin Hachuła, Michał Kosowski, Marcin Basiak, Bogusław Okopień

**Affiliations:** Department of Internal Medicine and Clinical Pharmacology, Medical University of Silesia, Medyków 18, 40-752 Katowice, Poland; mkosowski@sum.edu.pl (M.K.); mbasiak@sum.edu.pl (M.B.); bokopien@sum.edu.pl (B.O.)

**Keywords:** MASLD, GLP-1, diabetes, obesity, FIB4, semaglutide, dulaglutide

## Abstract

Metabolic Dysfunction-associated Steatotic Liver Disease (MASLD) is associated with the excessive collection of lipids in hepatocytes. Over 75% of diabetes patients typically have MASLD, and, at the same time, the presence of MASLD increases the risk of diabetes by more than two times. Type 2 diabetes and MASLD are independent cardiovascular disease (CVD) risk factors. New diabetes treatment should also take into account pleiotropic effects that reduce cardiovascular risk. The aim of our study is to investigate whether analogs of GLP1 receptors have a pleiotropic metabolic effect and global impact to decrease cardiovascular risk, and also reduce the risk of hepatic fibrosis in patients with MASLD. This study involved 41 patients with diabetes and dyslipidemia who also had atherosclerotic plaque and hepatic steatosis verified by ultrasonography and who were eligible to begin one of the GLP1 receptor agonists treatments. We observed a statistically significant decrease in: BMI (*p* < 0.001) waist and hip circumference (*p* < 0.001), glycated hemoglobin (*p* < 0.001) and creatinine (*p* < 0.05). Additionally, we obtained a decrease in FIB-4 (*p* < 0.001) and in the De Ritis (AST/ALT aminotransferase ratio) (*p* < 0.05). The positive correlation between the FIB-4 value and BMI, WHR, waist circumference and the De Ritis index was observed. In conclusion, semaglutide and dulaglutide had a beneficial effect on metabolic and cardiovascular risk factors in patients with type 2 diabetes. These medications had a positive effect on MASLD biochemical markers.

## 1. Introduction

Nonalcoholic Fatty Liver Disease (NAFLD) is associated with the excessive collection of lipids in hepatocytes. Worldwide, NAFLD occurs in one-third of the adult population and is firmly connected to metabolic dysfunctions, like obesity, dyslipidemia, and especially type-2 diabetes mellitus (T2DM). The prevalence of NAFLD has increased over 50% in recent decades. This rapid growth is caused by the pandemic of obesity and type 2 diabetes [1,2]. Considering that nine of ten obese and over 75% of T2DM patients typically have NAFLD and that at the same time, the presence of NAFLD increases the risk of diabetes by more than 2 times, it could be assumed that these disorders are comorbid and their underlying mechanisms are similar. The relationship between T2DM and NAFLD is bidirectional [3,4,5]. In 2023 a consensus was published on changing the name to Metabolic Dysfunction-associated Steatotic Liver Disease (MASLD) [6].

The mechanism of MASLD development is complex and consists of many factors. Nowadays, the prevailing view of the disease’s development is the “multi-hit hypothesis”. The most important component of this theory is insulin resistance (IR), which also plays a crucial role in the development of type 2 diabetes [7]. In hepatic cells, hyperinsulinemia stimulates fatty acid synthesis de novo; in turn, in adipose tissue, there is no inhibition of lipolysis, which increases the amount of free fatty acids in the liver. The results of these biochemical processes cause an increase in the deposition of triglycerides in the liver parenchyma and lead to the development of steatosis and, thus, “toxic” levels of fatty acids and other lipid metabolites. These induce mitochondrial dysfunction with oxidative stress, starts local inflammation, and lead to nonalcoholic steatohepatitis (NASH) [8]. Available data from the literature indicates that NASH is 7–10 times more common in patients with type 2 diabetes, and there is also an over double higher risk of hepatocellular carcinoma associated with MASLD in diabetes [9]. A recent meta-analysis by Ciardullo S et al. showed that up to 20% of patients with type 2 diabetes had signs of liver fibrosis in the elastographic examination [10]. 

In 2016, the European Association for the Study of Diabetes (EASD) and the European Association for the Study of Obesity (EASO) recommended screening for MASLD in patients with type 2 diabetes and looking for inaccurate glucose fasting in patients with MASLD [11]. However, there are no available simple and effective screening tools yet. Liver biopsy with histological assessment of MASLD is still the diagnostic gold standard in, but it is difficult to evaluate each patient [12]. There are many helpful scales to non-invasively diagnose MASLD, e.g., the Fatty Liver Index (FLI) or Lipid Accumulation Product (LAP) [13]. The next step is to confirm the diagnosis by using imaging tests, e.g., abdominal USG to assess the risk of liver fibrosis. For this purpose, the Fibrosis-4 (FIB-4) score seems to be the most useful in primary care. The algorithm is based on a few basic and widely available laboratory tests. FIB-4 has good sensitivity to exclude fibrosis with a negative predictive value over 90% [14]. Its usefulness was confirmed in the STELLAR trials, in which the results were compared with that from a biopsy [15]. Furthermore, a change in FIB-4 value may be used to assess the clinical progression of the disease [16]. 

Type 2 diabetes and MASLD are independent cardiovascular disease (CVD) risk factors. CVD is a major cause of mortality among patients with diabetes [17]. Also in MASLD patients, CVD one of the main causes of death overall, not only due to atherosclerosis progression but also due to the development of heart failure and arrhythmias [18,19,20]. In T2DM patients, MASLD doubles the risk of CVD compared to patients with diabetes but without liver dysfunction [21].

Considering the above information, the appropriate approach to the modern therapy of civilizational diseases, such as type 2 diabetes, should be to use treatments that affect not only the level of glycemia in the plasma, but also pleiotropically affect other cardiovascular risk factors. New hypoglycemic drugs, namely Glucagon-Like Peptide 1 receptor agonists (GLP-1RA), mimic the action of the Glucagon-Like Peptide-1 hormone, which is secreted from enteroendocrine cells or intestinal L cells. It belongs to the family of incretin hormones, which are responsible for the glucocorticoid effect in the body. GLP-1 receptors are located throughout our bodies; therefore, numerous studies have shown numerous pleiotropic effects connected with the therapy [22,23].

The aim of our study is to investigate whether of GLP1 receptor agonist have a pleiotropic metabolic effect and global impact to decrease cardiovascular risk and also reduce the risk of hepatic fibrosis in patients with MASLD.

## 2. Results

### 2.1. Study Group Characteristics

The study group consisted of 41 patients with a mean age of 60.5 ± 10 years, including 21 women. Overall, 25% were overweight (BMI > 25 kg/m^2^) and 68% were obese (BMI > 30 kg/m^2^). All subjects were diagnosed with type 2 diabetes mellitus (mean HbA1c: 8.7%; average duration time is 10 years) and dyslipidemia. The concomitant diseases included hypertension (80%), chronic kidney disease (17%—all in stage G3a), hypothyroidism (14%) and heart failure with reduced ejection fraction (9%). For diabetes treatment, patients chronically received metformin (97%), sulfonylurea (49%), DPP-4 inhibitors (10%), SGLT2 inhibitors (20%) and insulin (24%). Treatment was not changed during the intervention. At baseline, serum levels of ALT and AST were 26 U/L and 28 U/L, respectively, and the total cholesterol was 166.4 mg/mL, LDL was 84 mg/dL and TG was 153 mg/dL. The Median SBP was 135 mmHg and the mean DBP was 83 mmHg. There were 19.5% active smokers and none of the patients abused alcohol. In total, 21 subjects (51%) met the WHO criteria for physical activity. 

### 2.2. Metabolic Effect after 180 Days of Treatment

**Figure 1:**
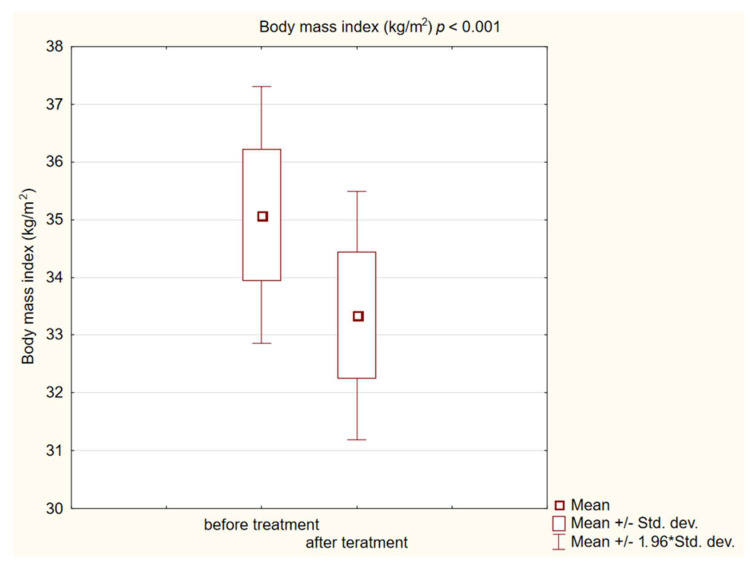
Body mass index (BMI) in study group before and after treatment. The “*” in “min/max 1.96 * stand. dev” is the standard record in statistica for data with normal distribution. Same for below figures.

**Figure 2:**
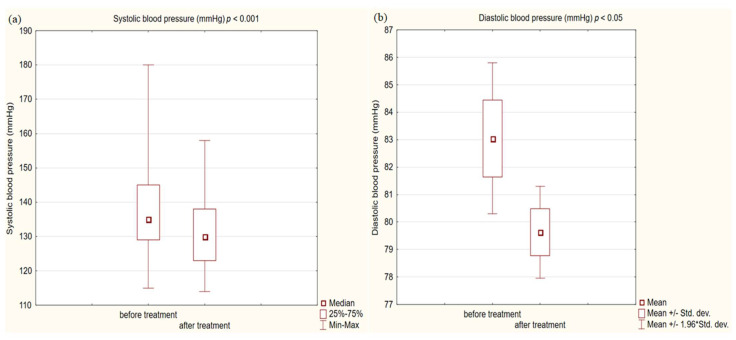
(**a**) Systolic blood pressure (SBP) in study group before and after treatment. (**b**) Diastolic blood pressure (DBP) in study group before and after treatment.

**Figure 3:**
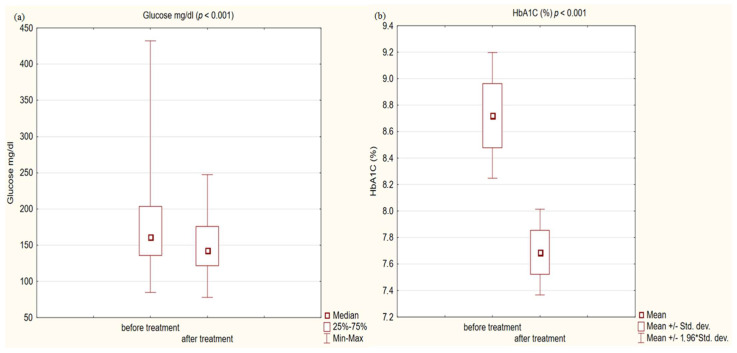
(**a**) glucose concentration in study group before and after treatment. (**b**) Glycated hemoglobin (HbA1C) concentration in study group before and after treatment.

**Figure 4:**
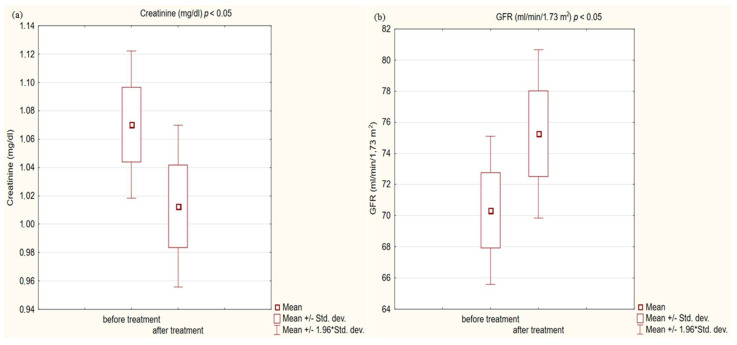
(**a**) Creatinine concentration in study group before and after treatment. (**b**) Glomerular filtration rate (GFR) in study group before and after treatment.

**Figure 5:**
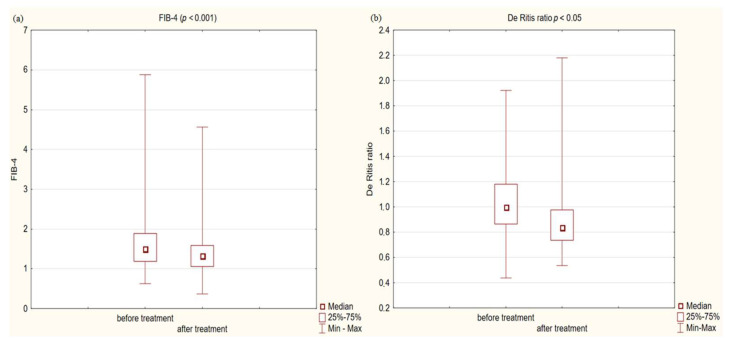
(**a**) Fibrosis-4 score (FIB-4) in study group before and after treatment. (**b**) De Ritis ratio in study group before and after treatment.

**Figure 6:**
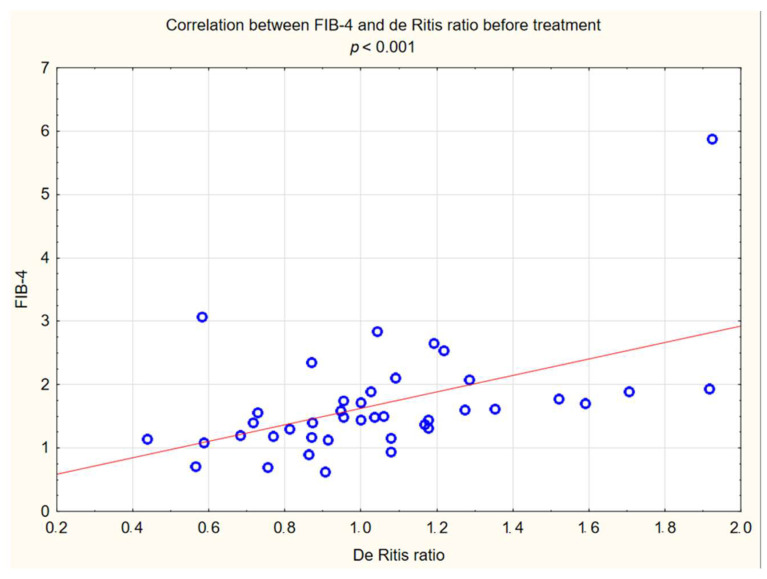
Correlation between FIB-4 and De Ritis ratio before treatment.

In the study group after treatment, we observed a statistically significant reduction in anthropometric parameters, including BMI (*p* < 0.001); on average, patients lost 4.9 kg of weight. There was also a significant decrease in waist and hip circumference (*p* < 0.001). Substantial differences in decreasing blood pressure for SBP (*p* < 0.001) and DPB (*p* < 0.05) were noted. In biochemical tests, a lower concentration of fasting glucose, glycated hemoglobin (mean: 7.69%, average reduction of 1.03%; *p* < 0.001) and creatinine (*p* < 0.05) were observed. Also, a statistically significant improvement of eGFR rate was observed (mean 4.93 mL/min/m^2^; *p* < 0.05). In terms of THE lipid profile, patients also benefited from a reduction in total cholesterol (TC), LDL fraction, TG and non-HDL cholesterol as well as an increase in HDL fraction; however, these changes were not statistically significant. More importantly, we obtained a statistically significant decrease in FIB-4 (*p* < 0.001). In the results for individual transaminases, we did not observe statistically significant differences, however, a decrease in the De Ritis ratio turned out to be statistically significant (*p* < 0.05). Detailed results are presented in Table 1 and Figure 1, Figure 2, Figure 3, Figure 4, Figure 5 and Figure 6.

### 2.3. Analysis of Correlations

The relationship between FIB-4 and some examined variables were verified. The positive correlation between the FIB-4 value and BMI, WHR and waist circumference were observed, and a strong correlation between the FIB-4 index and the de Ritis index was also observed (Figure 6. However, we have not observed a correlation between FIB-4 and any of the biochemical markers that were examined. Detailed results are presented in Table 2. 

### 2.4. Safety and Adverse Events

A total of 26 patients (63%) reported adverse effects. They mostly concerned the gastrointestinal system: 29% reported a feeling of fullness; 27%, nausea; and 19%, diarrhea. No serious adverse events were reported during this study. Only 14% of patients described the above-mentioned side effects as impairing normal functioning. In total, 2 patients discontinued therapy due to adverse events.

## 3. Discussion

In industrialized societies, cardiovascular diseases (CVD), such as coronary artery disease (CAD), peripheral artery disease (PAD), and cerebrovascular disease, are among the leading causes of death. According to the WHO, approximately 18 million people died of cardiovascular disease in 2019, accounting for one-third of all deaths worldwide [24]. Cardiovascular diseases are accelerated by metabolic diseases, such as obesity, metabolic-associated fatty liver disease, and diabetes, which increase their incidence and mortality [17,20,25]. It is a well-established fact that polypharmacy can result in medication nonadherence [26]. Therefore, modern patient-centered medicine should consider all metabolic and cardiovascular disease risk factors. Attempts should be made to implement treatments that, in addition to their primary objective, such as lowering the level of glycated hemoglobin, will influence other components of the metabolic syndrome, thereby lowering cardiovascular risk. By reducing the number of pills used, compliance could be improved, hence decreasing the cardiovascular risk [27]. In clinical trials, semaglutide and dulaglutide have shown cardiovascular disease mortality, nonfatal heart attacks and strokes in diabetic patients reduction [28,29].

MASLD and its repercussions are becoming a global health problem, therefore the necessity for available and rapid diagnostic capabilities in primary care. Rungta S. et al. proved in their study that FIB-4 should be the preferred non-invasive fibrosis test, even before the Fibroscan [30]. A systematic review carried out by Lee J. et al. showed that FIB-4, could be used to stratify the risk of liver-disease-related morbidity and mortality [31]. Among the non-invasive markers of fibrosis, FIB 4 best classified fibrosis compared to the histopathological result [32]. The De Ritis index is the ratio of the plasma concentration of aspartate aminotransferase to alanine aminotransferase. In chronic hepatic disease, such as MASLD, an elevated AST/ALT ratio is predictive of future complications, such as fibrosis followed by cirrhosis [33]. In the case of MASLD’s progression to fibrosis, increased mitochondrial damage leads to the release of the mitochondrial AST fraction [34]. It is expected that elevated AST and De Ritis scores in the course of liver disease are associated with a higher risk of CVD and a worse outcome [35]. Ndrepepa G. et al. in their work confirmed that an increased De Ritis ratio predicts 3-year all-cause, cardiac and noncardiac mortality. Their risk models showed that for each unit of a higher De Ritis index, the adjusted risk of mortality increases by 24% [36]. It is important to emphasize that the De Ritis index has weaknesses, one of the main ones being that in the case of a proportional increase in AST and ALT, the De Ritis index may vary slightly and therefore cannot reveal changes (or risks) associated with abnormal aminotransferase levels. 

In our interventional study we obtained a statistically significant decrease in FIB-4 (*p* < 0.001) from 1.5 to 1.33 after 180 days using Glucagon-Like Peptide-1 receptor agonists with a typical hypoglycemic dose. We obtained a decrease in the De Ritis ratio from 1 to 0.84 that turned out to be statistically significant (*p* < 0.05). We did not observe statistically significant differences in the results of individual transaminases; however, a level of aspartate aminotransferase (AST) decreased with almost statistical significance (*p* = 0.06) was observed. We assume that the decrease in aminotransferases would be significant in the case of a larger research group. In conjunction with other factors, this may indicate stopping the progression of liver disease. Our findings are consistent with other scientific studies involving patients diagnosed with diabetes. Liraglutide has a well-established position in research confirming the effect on improving parameters in patients with fatty liver. There is evidence of improvement in terms of histological fatty liver in a multicenter, double-blind, randomized, placebo-controlled phase 2 study [37], in which reduced insulin resistance and hepatic lipogenesis, which are crucial for the development of fatty liver [38,39], decreased in aminotransferase serum level ALT and AST [38], and a reduction in the content of hepatic adipose tissue were measured by diagnostic imaging methods [39,40,41,42]. Newsome et al., in their multicenter, randomized, double-blind, placebo-controlled, parallel-group phase 2 trial, showed that semaglutide use was significantly related to the higher percentage of patients with NASH symptom remission proven by liver biopsy compared to the placebo group. Participants in this study also had a decrease in aminotransferase levels [43]. On the other hand, the latest study conducted in 2023 in a small group of patients with NASH or cirrhosis taking semaglutide did not confirm histological improvement after 48 weeks, but improvements were seen in non-invasive markers and a clinically significant reduction in hepatic steatosis assessed by MRI was reported [44]. Differences between histological results in these studies may be connected to the study duration, 72 weeks vs. 48 weeks, and the patients in the Loomba R et al. study were in a more advanced stage of the disease, which may indicate a more-difficult-to-reverse process. Subcutaneously administered semaglutide reduced hepatic steatosis on MRI in a 48-week study [45]. Dulaglutide in the D-LIFT study (dulaglutide on liver fat) investigating the effect on liver fat content (as examined by magnetic resonance imaging) proved the significant extenuation in liver fat volume [46]. Finally, a meta-analysis conducted by Mantovani A et al. confirmed the effectiveness of various GLP1 receptor agonist in reducing the percentage of hepatic fat [47]. Also Kovalic AJ et al. in their meta-analysis proved that semaglutide is one of the most effective drugs to treat NAFLD, which was confirmed by elastography and non-invasive blood tests [48]. The same results were published in 2023 by Gu Y et al. [49] Similar results to our study on FIB-4 decrease were obtained by Arai, T et al. in their pilot study for oral semaglutide treatment [50]. Also, Carretero-Gómez J et al. obtained a statistically significant decrease in the FIB-4 value [51]. In the AWARD study, dulaglutide significantly reduced aminotransferases levels compared to a placebo, especially in patients with NAFLD [52]. Dutta et al. in a meta-analysis showed a significant decrease in the level of aminotransferases and radiological features of fatty liver after semaglutide treatment [53]. Contradictory, no statistically significant decrease in aminotransferases was observed in this interventional study [46] after treatment with dulaglutide. Considering the difficulties of interpretation associated with the variability of aminotransferase levels and the divergences in the available literature regarding the De Ritis index, its use in forecasting of MASLD patients should be combined with other parameters. In our study, we noted a decrease in the De Ritis index, which was strongly correlated with the FIB4 value (R: 0.54 *p* < 0.001), which may indicate its usefulness. Therefore, further research is needed in this area. Considering the above data, the effect of GLP1-RA on the improvement of liver function in patients with MASLD is confirmed and strongly established in the literature via numerous data, histopathological results and imaging tests. In our work, we have shown that basic biochemical tests and algorithms can be useful for monitoring the response to treatment in primary care.

The pillar of MASLD treatment is weight loss and the control of other metabolic syndrome risk factors, such as central obesity, hyperglycemia, dyslipidemia, and hypertension. We observed improvements in all traditional cardiovascular risk factors, including weight, BMI, waist circumference, blood pressure, plasma glucose level, and lipid profile.

One of the most connected cardiovascular risk factors in the metabolic syndrome group is obesity, especially visceral obesity. Obesity causes insulin resistance and is also a main risk factor for MASLD [4]. Our study showed that the use of semaglutide or dulaglutide in hypoglycemic dose causes a weight loss average of 4.9 kg. What is important is that our patients during the study did not change their diet or physical activity. We obtained a statistically significant decrease in BMI, hip, and waist circumference (*p* < 0.001). Despite a statistically significant decrease in BMI at the end of the intervention, 65.8% of patients in our group remained obese (BMI > 30 kg/m^2^). This is probably due to the use of hypoglycemic doses of GLP1-RA, which are lower than the doses used in the treatment of obesity. In addition, the intervention time was short. Furthermore, the positive correlation between the FIB-4 value and BMI, WHR and waist circumference was observed. The effect of GLP1 receptor analogs on obesity is well known in diabetic and non-diabetic patients. As a class of antihyperglycemic medicine, GLP-1RAs were associated with notable weight loss between 1.5 to 4.3 kg [28,29,54,55]. Our findings are extremely similar. Currently, semaglutide and liraglutide preparations are approved for the treatment of obesity without diabetes [56,57].

Interestingly, in terms of lipid profile, our patients reduced their total cholesterol, LDL, TG, non-HDL cholesterol and increased their HDL, but these changes were not statistically significant in our study. Numerous studies confirm the beneficial effect of semaglutide and dulaglutide on the lipid profile. Large randomized studies have shown a beneficial effect, namely lowering cardiovascular risk, among others, through changes in the values of the lipid profile [28,29]. Differences in the statistical significance of the decrease in the lipid profile value may be due to the fact that in the SUSTAIN-6 study, 72.6% of patients were treated with a statins, and in the REWIND study, it was 66%, compared to our group, where each patient was treated with statins. Additionally, in the REWIND study, baselines for the values of individual fractions were higher than in our group, e.g., LDL 99 mg/dL vs. 84 mg/dL in our group. Moreover, at the time of the initiation of the glp1 analogue into therapy, 51% of our patients met the alignment criteria for LDL values according to the AHA and ESC guidelines. To sum up, we assume that in the enrollment, our patients had better-balanced lipid metabolism, which contributed to a less significant decrease in values during therapy. Kuchay MS et al. and Arai T et al., in their intervention studies, which was similar to our work, reported improvement in lipid profile, however without statistical significance [46,50]. 

We also received a satisfactory improvement in other typical cardiovascular risk factors, such as blood pressure or fasting glucose and the metabolic control of glucose expressed as a percentage of glycated hemoglobin. The decreases obtained were so noteworthy that we obtained statistical significance at the level (*p* < 0.001) in all these issues. On average, in terms of systolic blood pressure, we obtained a decrease of 5 mmHg, and HbA1c was reduced by 1.03%. Despite such a significant decrease in glycemia, only 37% of patients achieved the criteria for glycemic control defined as HbA1c < 7% according to the ADA guidelines [58]. The head-to-head analysis of phase 3 clinical trials showed a significant decrease in glycated hemoglobin levels for each GLP1-RA [59].

Kidney function was the last measure we analyzed. Chronic kidney disease (CKD) is not a component of the metabolic syndrome, but it is a well-established fact that CKD is strongly associated with cardiovascular risk [60]. Diabetes is the leading cause of chronic kidney disease, accounting for over 40 percent of all new cases worldwide [61]. Obesity and abnormal blood pressure also contribute to the cause [62]. To effectively manage diabetes and kidney disease, which are comorbidities, it is necessary to use drugs with nephroprotective properties in addition to its hypoglycemic activity. There are SGLT2 receptor inhibitors currently available with clinically proven protective effects on kidney function [63,64,65]. The GLP1-RA, due to their beneficial effect on numerous CKD risk factors, are of interest to scientists as drugs with nephroprotective potential. In our small study, we achieved a statistically significant decrease in creatinine (*p* < 0.001) and an increase in glomerular filtration by an average of 4.93 mL/min/1.73 m^2^ (*p* < 0.05). Our research group consists mainly of subjects without diagnosed CKD; only 17% of patients had a reduced eGFR, but all were in the G3a stage of CKD. The outcomes given by us are consistent with the available literature, which confirms the nephroprotective potential of this group of drugs in studies involving a larger number of participants. The post hoc analysis of the SUSTAIN 6 and LEADER trials showed that semaglutide and liraglutide in patients with type 2 diabetes offered kidney-protective effects [66]. In a series of other large STEP studies, semaglutide in high dose in non-diabetic obese patients with high baseline eGFR also improved kidney function by reducing UACR levels [67]. At the hypoglycemic dose, semaglutide significantly reduced UACR levels [66]. Also, dulaglutide in patients with diabetes improved kidney function by reducing the decrease in glomerular filtration [29,68]. There is no knowledge on to what extent the beneficial effect on renal function is due to concomitant changes in glycated hemoglobin, body weight, and blood pressure, and to what extent other renal protective mechanisms exist. A clinical trial, REMODEL, is currently underway to accurately assess the effect of semaglutide on kidney function.

However, this study has several limitations. Firstly, our study group is small. Secondly, this study concerned only patients from the Upper Silesia region of Poland; the obtained results could be different due to living place, race, and environmental factors. Thirdly, while planning the study, we planned to use only semaglutide, but problems with supply and availability on the Polish market forced us to change the tested drug to dulaglutide during the study. The other important limitation of our study was a lack of a control group treated with placebo or an active comparator. Lastly, renal function was assessed retrospectively; the results obtained could be affected by other factors, and we did not examine microalbuminuria, which is a better marker of the early stage of CDK in patients with diabetes.

## 4. Materials and Methods

### 4.1. Study Population

**Figure 7:**
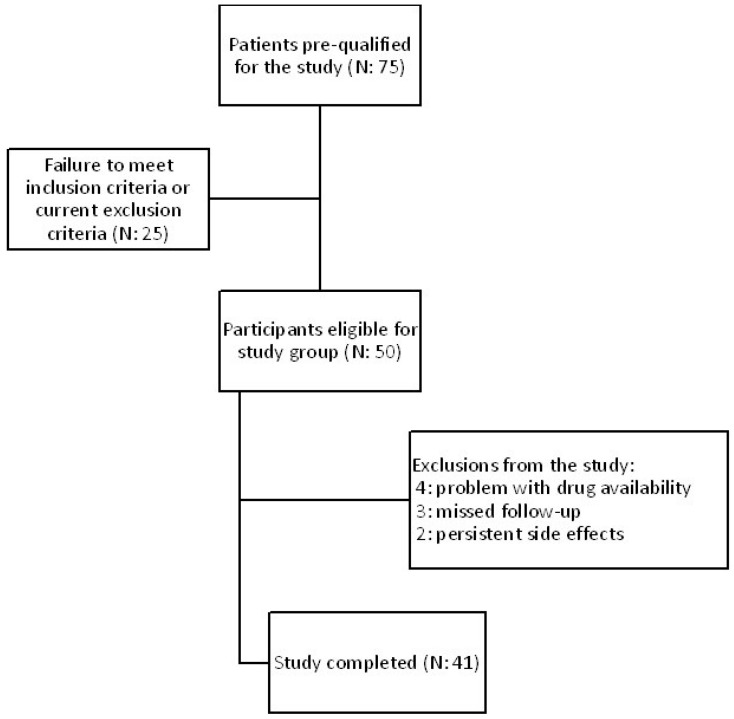
Flowchart of the study.

41 patients aged 41–81 (mean: 60) out of 75 completed the study. All participants were diagnosed with type 2 diabetes mellitus, dyslipidemia, confirmed atherosclerosis based on B-mode ultrasound common carotid intima-media thickness, and hepatic steatosis as determined by abdominal ultrasonography. The medical experiment was performed in the years January 2022–May 2023. Subjects who fulfilled all the very detailed and narrow inclusion and exclusion criteria were eligible for study entry. Each patient gave their informed consent in accordance with the Declaration of Helsinki. All the information about the subjects was anonymized. Patients were recruited at the Department of Internal Medicine and Clinical Pharmacology in Katowice, Poland, and as referrals from the Mysłowice and Imielin diabetes outpatient departments. The study protocol was approved by the Bioethical Committee of the Medical University of Silesia PCN/CBN/0052/KB1/45/I/22. All included subjects were treated with one of GLP1 receptor agonists, either semaglutide (*n* = 16) or dulaglutide (*n* = 25), at a typical hypoglycemic dose and administered every week at the same time of the day. The choice of treatment was determined by the drugs availability on the Polish market. During the intervention, the GLP1 analogue therapy was not modified. The therapeutic intervention lasted 180 days. Figure 7 shows the flowchart of the study.

### 4.2. Inclusion and Exclusion Criteria

Type 2 diabetes; dyslipidemia, defined as plasma total cholesterol (TC) > 200 mg/dL and/or triglycerides (TG) > 150 mg/dL; the presence of atherosclerotic plaque in the common carotid artery, confirmed by ultrasound examination; and hepatic steatosis, confirmed by abdominal ultrasonography examination, were the inclusion criteria. 

Patients were excluded from the study in the following cases: pregnancy and breastfeeding period; type 1 diabetes; uncompensated thyroid disease; chronic pancreatitis; the acute exacerbation of autoimmune disorders; alcoholism; any acute and chronic inflammatory processes, including COVID-19 infection 4 weeks before inclusion of the study; chronic kidney disease in stage below G3b, with eGFR < 45 mL/min/1.73 m^2^; acute and chronic liver diseases expressed as an increase in transaminases above 3 times the norm; or diagnosed chronic viral hepatitis in medical history. Furthermore, cardiac disorders, like the exacerbation of chronic heart failure and unstable coronary artery disease, a history of percutaneous coronary intervention (PCI), coronary artery bypass grafting (CABG) or stroke less than 3 months before starting the study were also reasons for exclusion. After the intervention, all of the subjects were interviewed. They were also excluded if within the last 6 months, they increased their physical activity, changed their type of diet, their treatment was modified, or started therapy with a new drug with a proven effect on lipid serum levels or with known pleiotropic effect (e.g., statins, fibrates, ezetimibe, niacin, non-selective beta-blockers, metformin, SGLT2 inhibitors, or ursodeoxycholic acid). Additionally, if they had a coronary or stroke incident or suffered a severe infection.

### 4.3. Laboratory and Anthropometric Measurements

All measurements were taken before study enrollment and after 6 months of treatment by a physician. Body weight and height were measured before following standard procedures, and body mass index (BMI) was calculated in kg/m^2^. Waist and hip circumferences were measured at the typical location, and the waist/hip ratio (WHR) was computed. Arterial blood pressure (BP) was measured twice in the sitting position in the arm without vascular access. For this purpose, the Omron M400 Intelli IT automatic device was used. To estimate the glomerular filtration rate (eGFR), the CKD-EPI formula was used. The values were presented in mL/min/1.73 m^2^. FIbrosis-4 was evaluated by math formula using the age, levels of aminotransferase, and platelet amount. Routine laboratory measurements were performed in the certificated laboratory, and venous blood samples were collected after an overnight 12 h fasting at 8 a.m. before the treatment and after 180 days of intervention.

### 4.4. Arteriosclerotic Plaque Examination

The examination of the carotid arteries and the assessment of complex intima media thickness (C-IMT) in the extracranial segment was performed using B-mode ultrasound with a linear probe at a frequency of 7.5–10 MHz on a Hitachi Aloka F37 ultrasound machine. According to the Atherosclerosis Risk in Communities Study (ARIC), the C-IMT was evaluated 3 times, and the mean score was taken into consideration. The measurement was performed in the distal common carotid (1 cm proximal to the carotid bulb). For confirmation of atherosclerotic plaque in the carotid artery, we assumed a thickness of the C-IMT complex > 1.5 mm or the presence of plaque, in accordance with the guidelines.

### 4.5. Hepatic Steatosis Examination

According to the EASL–EASD–EASO Clinical Practice Guidelines, abdominal ultrasound examination was used to confirm MASLD. All examinations were performed using a Hitachi Aloka F37 ultrasound machine with a curved probe at a frequency of 2–6 MHz. To recognize hepatic steatosis, hepatic/renal echo intensity ratio (H/R) was used. H/R ratio is defined as an evident ultrasonographic contrast between the hepatic parenchyma and the right renal cortex. These images were assessed with both the liver and right kidney clearly visualized and were obtained in the right intercostal space in the midaxillary line. Secondary causes of hepatic steatosis; like overuse of alcohol; drugs, e.g., amiodarone and methotrexate; and hepatotropic viruses; were excluded.

### 4.6. Statistical Analysis

The data was processed using Statistica TIBCO Software Inc. (2017) version 13.3 software (Palo Alto, CA, USA), which was licensed by the Medical University of Silesia in Katowice. To assess the normality of distributions, we used the Shapiro–Wilk test. Values were presented as means and 95% confidence intervals or medians with Q1–Q3 values. To compare quantitative variables, the *t*-test for dependent means was used. Also, we used the Wilcoxon test in the case of non-compliance with the condition of the *t*-test. We also used Spearman rank correlation to assess the relationship between variables. We assumed a *p*-value of less than 0.05 was statistically significant.

## 5. Conclusions

In our research, we confirmed that GLP1 receptor agonists have a beneficial metabolic effect on cardiovascular risk reduction. In conclusion, semaglutide and dulaglutide had a beneficial effect on metabolic and cardiovascular risk factors in patients with type 2 diabetes. These medications had a positive effect on MASLD biochemical markers. In addition, they caused weight loss, decreased waist circumference, blood pressure, and enhanced kidney function.

## Figures and Tables

**Table 1:** Effect of Glucagon-Like Peptide-1 (GLP-1) agonists on metabolic parameters. BMI—body mass index; WHR—waist/hip ratio; DBP—diastolic blood pressure; HbA1C—glycated hemoglobin; GFR—glomerular filtration rate; SBP—systolic blood pressure; TC—total cholesterol; LDL—low-density lipoprotein cholesterol; HDL—high-density lipoprotein cholesterol; non-HDL—non-high-density lipoprotein cholesterol; TG—triglycerides; AlAT—alanine transaminase; AspAT—aspartate transaminase; GGTP—gamma-glutamyl transpeptidase; FIB-4—fibrosis-4 score; SD—standard deviation; Q1—first quartile; Q3—third quartile after treatment.

	Study Group before Treatment			Study Group after 180 Days of Treatment			
	Mean	SD		Mean	SD		*p*
BMI (kg/m^2^)	35.08	7.26		33.34	7.01		<0.001
WHR	0.97	0.06		0.97	0.07		0.087
Waist circumference (cm)	115.5	17.1		111.5	15.5		<0.001
Hip circumference (cm)	118.5	15.5		115.3	14.2		<0.001
DBP (mmHg)	83.04	8.98		79.63	5.47		<0.05
HbA1C (%)	8.72	1.55		7.69	1.06		<0.001
GFR (ml/min/1.73 m^2^)	70.34	15.55		75.27	17.67		<0.05
Creatinine (mg/dL)	1.07	0.17		1.01	0.19		<0.05
	Median	Q1	Q3	Median	Q1	Q3	
SBP (mmHg)	135	129	145	130	123	138	<0.001
Glucose (mg/dL)	161	135.5	203.3	143	121.8	176.1	<0.001
TC (mg/dL)	166.4	146.3	198.6	158.1	144.1	192.6	0.34
LDL (mg/dL)	84	67	97	80	57.2	115	0.32
HDL (mg/dL)	49	43	54.7	51.3	46.3	60.5	0.2
non-HDL (mg/dL)	112.2	100	137	104.7	88.8	137.4	0.13
TG (mg/dL)	153	108.8	192	144	104	181.8	0.36
De Ritis Ratio (AspAT/AlAT)	1	0.86	1.18	0.84	0.74	0.96	<0.05
AlAT (U/L)	26	22	39	30	23	45	0.68
AspAT (U/L)	28	23	40	25	20.4	40	0.06
GGTP (U/L)	38	30	54	37	23	49	0.29
FIB-4	1.5	1.19	1.89	1.33	1.06	1.59	<0.001

**Table 2:** Correlation between fibrosis-4 (FIB-4) score and metabolic parameters.

	FIB-4 Score
Body mass index	R = 0.34, *p* < 0.05
Waist/hip ratio	R = 0.41, *p* < 0.01
Waist circumference	R = 0.42, *p* < 0.01
De Ritis ratio	R= 0.54, *p* < 0.01
Glycated hemoglobin	R = 0.057, *p* > 0.05
Glomerular filtration rate	R = −0.11, *p* > 0.05
Total cholesterol level	R = −0.11, *p* > 0.05
Low-density lipoprotein cholesterol level	R = −0.102, *p* > 0.05
High-density lipoprotein cholesterol level	R = 0.008, *p* > 0.05
Non-high-density lipoprotein cholesterol level	R = −0.162, *p* > 0.05
Triglycerides level	R = −0.067, *p* > 0.05

## Data Availability

Data is contained within the article.
